# Author Correction: Tracing two decades of carbon emissions using a network approach

**DOI:** 10.1038/s41598-024-59779-w

**Published:** 2024-04-17

**Authors:** Gianluca Guidi, Rossana Mastrandrea, Angelo Facchini, Tiziano Squartini, Christopher Kennedy

**Affiliations:** 1 IMT School for Advanced Studies, Piazza San Francesco 19, 55100 Lucca, Italy; 2 Department of Computer Science, University of Pisa, Largo Bruno Pontecorvo 3, 56127 Pisa, Italy; 3 Institute for Advanced Study (IAS), University of Amsterdam, Oude Turfmarkt 145, 1012 GC Amsterdam, The Netherlands; 4 Institute for Integrated Energy Systems, University of Victoria, Victoria, BC Canada

Correction to: *Scientific Reports* 10.1038/s41598-024-57351-0, published online 27 March 2024

The original version of this Article contained an error in the Acknowledgements section.

“This work is supported by the European Union—Horizon 2020 Program under the scheme ‘INFRAIA-01-2018-2019—Integrating Activities for Advanced Communities’, Grant Agreement n. 871042, ‘SoBigData++: European Integrated Infrastructure for Social Mining and Big Data Analytics’ (http://www.sobigdata.eu). This work is also supported by the project ‘Network analysis of economic and financial resilience’, Italian DM n. 289, 25-03-2021 (PRO3 Scuole) CUP D67G22000130001. RM acknowledges support from the ‘Programma di Attività Integrata’ (PAI) project ‘Prosociality, Cognition and Peer Effects’ (Pro.Co.P.E.), funded by IMT School for Advanced Studies Lucca. RM is member of the INDAM group “Gruppo Nazionale per l’Analisi Matematica, la Probabilità e le loro Applicazioni (GNAMPA)”.”

now reads:

“This work is supported by SoBigData.it. SoBigData.it receives funding from European Union – NextGenerationEU – National Recovery and Resilience Plan (Piano Nazionale di Ripresa e Resilienza, PNRR) – Project: “SoBigData.it – Strengthening the Italian RI for Social Mining and Big Data Analytics” – Prot. IR0000013 – Avviso n. 3264 del 28/12/2021. This work is also supported by the project ‘Network analysis of economic and financial resilience’, Italian DM n. 289, 25-03-2021 (PRO3 Scuole) CUP D67G22000130001. RM acknowledges support from the ‘Programma di Attività Integrata’ (PAI) project ‘Prosociality, Cognition and Peer Effects’ (Pro.Co.P.E.), funded by IMT School for Advanced Studies Lucca. RM is member of the INDAM group “Gruppo Nazionale per l’Analisi Matematica, la Probabilità e le loro Applicazioni (GNAMPA)”.”

The original Article has been corrected.

